# Expression, Subcellular Localization, and Interactions of CPK Family Genes in Maize

**DOI:** 10.3390/ijms20246173

**Published:** 2019-12-06

**Authors:** Muhammad Hayder Bin Khalid, Muhammad Ali Raza, Hao Qiang Yu, Imran Khan, Fu Ai Sun, Ling Yang Feng, Jing Tao Qu, Feng Ling Fu, Wan Chen Li

**Affiliations:** 1 Maize Research Institute, Sichuan Agricultural University, Chengdu 611130, China; haider2323@gmail.com (M.H.B.K.); yhq1801@sicau.edu.cn (H.Q.Y.); andapanda86@outlook.com (F.A.S.); pandaanda86@outlook.com (J.T.Q.); 2 College of Agronomy, Sichuan Agricultural University, Chengdu 611130, China; razaali0784@yahoo.com (M.A.R.); lilymalik87@hotmail.com (L.Y.F.); 3 Department of Grassland Science, Sichuan Agricultural University, Chengdu 611130, China; imran.62k@gmail.com

**Keywords:** calcium dependent protein kinases, PP2C, SnRK, MAPK, ABA, signal transduction, Y2H, BiFC

## Abstract

Calcium-dependent protein kinase (CPKs) is a key player in the calcium signaling pathway to decode calcium signals into various physiological responses. cDNA sequences of 9 *Zm*CPK genes were successfully cloned from all four phylogenetic groups in maize. qRT-PCR analysis showed the expression variation of these selected genes under abscisic acid (ABA) and calcium chloride (CaCl_2_) treatment. Due to the presence of N-myristoylation/palmitoylation sites, the selected *Zm*CPK members were localized in a plasma membrane. To clarify whether *Zm*CPK, a key player in calcium signaling, interacts with key players of ABA, protein phosphatase 2Cs (PP2Cs) and the SNF1-related protein kinase 2s (SnRK2s) and mitogen-activated protein kinase (MAPK) signaling pathways in maize, we examined the interaction between 9 CPKs, 8 PP2Cs, 5 SnRKs, and 20 members of the MPK family in maize by using yeast two-hybrid assay. Our results showed that three *Zm*CPKs interact with three different members of *Zm*SnRKs while four *Zm*CPK members had a positive interaction with 13 members of *Zm*MPKs in different combinations. These four *Zm*CPK proteins are from three different groups in maize. These findings of physical interactions between *Zm*CPKs, *Zm*SnRKs, and *Zm*MPKs suggested that these signaling pathways do not only have indirect influence but also have direct crosstalk that may involve the defense mechanism in maize. The present study may improve the understanding of signal transduction in plants.

## 1. Introduction

Plants suffer from various environmental stresses, including drought, high salinity, and extreme temperatures [1,2,3]. To cope with these stresses, plants have developed sophisticated signal transduction pathways and a series of survival mechanisms. Among those pathways and mechanisms, transitory calcium is an important messenger of signal transduction that responds to hormonal and biotic or abiotic stresses [4,5,6]. Calcium signals are deciphered by calcium sensors, including calcium dependent protein kinases (CPKs). Genetics and biochemical studies have shown that CPKs are the key players in biological processes and plant signaling pathways, including innate immunity, oxidative burst, abiotic stress responses, and hormone signaling [7,8,9,10,11,12,13,14,15].

Abscisic acid (ABA) signaling pathway regulate many processes of plant growth and development under abiotic stress conditions, such as dehydration or high salinity [3,16,17]. Initially, it was thought that Ca^2+^- and ABA-mediating signaling pathways were independent of each other [18,19], or that the latter regulates the former [20], but later it was found that these two pathways are interdependent [21,22,23,24]. Phosphorylation of two ABA-responsive transcription factors (ABF1 and ABF4) by AtCPK4, 11, and 32, suggested the role of kinases in the regulation of ABA signaling through these transcription factors under stress conditions [11,25]. *AtCPK3*, 6, 10, 21, and 23 of the *Arabidopsis* CPK family control stomatal closure by two signaling pathways of ABA and Ca^2+^ [26,27,28,29]. In maize leaf protoplasts, two CPKs are able to transactivate the expression of an ABA-inducible gene in the absence of the hormone [21]. In seed germination and seedling growth of *Arabidopsis*, ABA sensitivity was enhanced by the overexpression of *ZmCPK4* [30]. The mutants of CPK1 and CPK1a inhibited the activity of protein phosphatases type 2Cs (PP2C), which is capable of blocking the responses to ABA in *Arabidopsis* [21]. All this evidence suggests that CPK plays a critical role at the intersection of ABA and Ca^2+^ signaling pathways [31]. *AtCPK4* and *AtCPK11* catalyze the ABF1 and ABF4 (ABRE2), and meanwhile, ABF1 is also a target of *SnRK2*.2 and *SnRK2*.3, indicating that two kinases can share a single substrate. On the other hand, MAPK signaling cascade is another pathway which proceeds beside calcium signaling and is triggered by the same environmental stimuli. Most of the MAPK family members in *Arabidopsis* are related to the pathogen response, while *At*CPKs were found to play their roles under abiotic stress as well as pathogen response [15,32]. MAPKs and CPKs are thought to be the key components arbitrating immunity in plants and are activated in response to almost the same stimuli [33,34,35,36]. Crosstalk between CPK and MAPK signaling pathways have been studied in animals, but have hardly been focused in plant systems [37,38]. Reports have suggested that MAPKs and CPKs act differentially in salt signaling and innate immunity without direct crosstalk [12,39]. In tobacco, it has been suggested that MAPK and CPK signaling mechanisms proceed in parallel, to provide backup and fine-tuning of the overlapping reactions underway [40]. With regard to hindrance in the expression of *ZmMPK5* due to *ZmCPK11* silencing, the role of *ZmCPK11* upstream of *ZmMPK5* has been proposed [41]. Phosphorylation of *OsMPK5* by *OsCPK18* proposed the direct crosstalk between CPK and MAPK signaling pathways [42].

**Figure 1:**
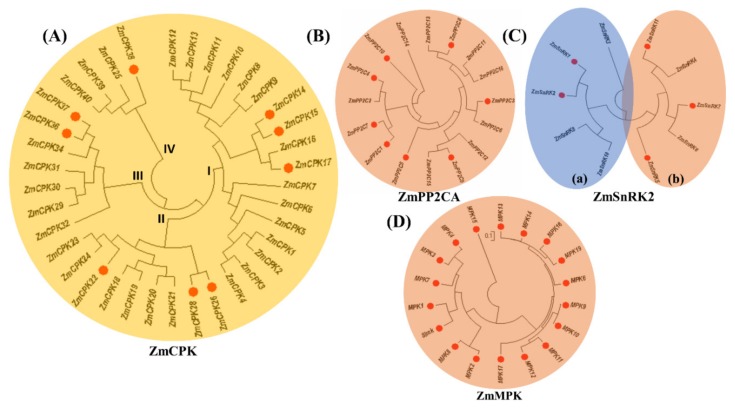
Phylogenetic tree of CPKs, PP2Cs, SnRKs, and MPKs from maize. The phylogenetic trees were constructed after alignment of full-length sequences of (**A**) ZmCPKs, (**B**) *Zm*PP2Cs clade A, (**C**) *Zm*SnRK2s, and (**D**) ZmMAPKs from maize using the neighbor-joining method. MEGA7 was used with 1000 bootstraps. Members marked in red were used in this study.

Although CPKs seem to play their role in ABA signaling, it is not clear whether there is a link between the CPK pathway and the core ABA signaling components in ABA signaling. Moreover, in addition to the classical MAPK cascade model, plants may have developed a calcium-mediated pathway for the activation of MAPK. Taking into account the importance of CPKs, we isolated nine CPKs distributed in four groups on the basis of phylogenetic analysis (Figure 1). Expression analysis of the selected CPKs under ABA and CaCl_2_ treatment explained their sensitivity towards these treatments. Because of the importance of location of protein expression, we visualized subcellular localization of selected members in tobacco leaves. Yeast two-hybrid assay revealed the interaction between *Zm*CPKs, *Zm*SnRKs, and *Zm*MPKs.

It has become clear that signaling pathways have to be envisaged as complex networks rather than as linear or branched signaling pathways.

## 2. Results

### 2.1. Expression Analysis of ZmCPK Family Members

**Figure 2:**
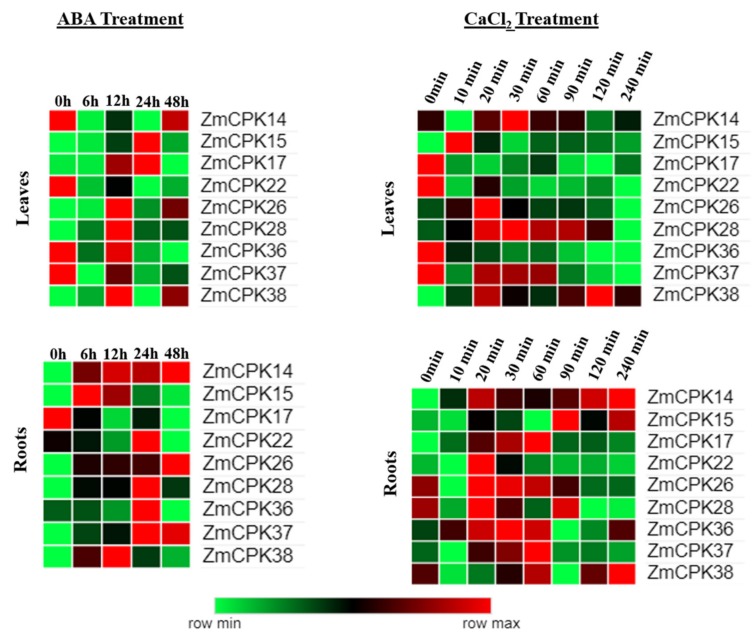
Heat maps illustrating the expression pattern of nine *ZmCPK* genes in maize B73 leaf and root under abscisic acid (ABA) and CaCl_2_ treatment. The *ZmGAPDH* gene was used as an internal control. Both leaf and root tissues were collected at time points of 0, 6, 12, 24, and 48 h after ABA and at 0, 10, 20, 30, 60, 90, 120, and 240 min after CaCl_2_ treatment.

Gene expression patterns can provide important information about gene function. In this study, the expression patterns of the nine *Zm*CPKs were detected by qRT-PCR, and the results showed variations under ABA and CaCl_2_ as calcium treatments. The analysis with qRT–PCR showed that the expressions of these nine *Zm*CPK genes were regulated by these treatments. Under ABA and calcium treatment, we noticed that *Zm*CPK expressed differentially at different time points (Figure 2). *ZmCPK14*, 22, 36, and 37 appeared to be significantly down-regulated at 6 h of post-ABA treatment relative to the control leaf samples. On the other hand, *ZmCPK14*, 15, and 38 seemed to be upregulated at 6 h of post ABA-treated root samples. Besides *ZmCPK14*, 15, and 22, all other genes were highly expressed at 12 h of ABA-treated leaf samples and expression of most of the genes was changed into downregulation at 24 h, i.e., *ZmCPK26*, 28, 36, 37, and 38. Unlike leaf samples, root samples had a downregulation of the *ZmCPK17*, 26, 28, and 36 genes. *ZmCPK37* and *ZmCPK38* genes were expressed differentially at 48 h in leaf and root samples after ABA treatment.

Moreover, the expression of nominated nine CPKs was evaluated both in leaves and roots at different time points under CaCl_2_ treatment. Compared to control samples of leaves, sudden expression change was observed in all selected genes at 10 min of post-CaCl_2_ treatment. However, the expressions of *ZmCPK17*, 22, and 36 were identified to be downregulated in CaCl_2_-treated leaves. Although the expression patterns of these nine genes were also evaluated under root samples. *ZmCPK14*, 17, 26, 36, and 37 had upregulation in the first hour, i.e., at 20, 30, and 60 min, in selected root samples. The different response of the transcripts of these *Zm*CPK genes to ABA and CaCl_2_ treatments suggested their potential role in resistance against abiotic stress. Given the expression pattern of the genes, all the genes had a heartbeat-like expression pattern at different time points. We can predict per expression levels of these genes that the first hour is most important after abiotic stress treatment, i.e., ABA and CaCl_2_, and these findings could be useful for researchers for detailed functional analysis.

### 2.2. Subcellular Localization of Selected ZmCPK Proteins

**Figure 3:**
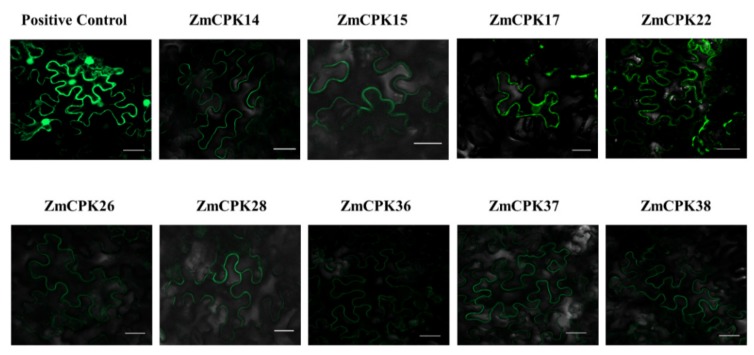
Subcellular localization of selected *Zm*CPK-GFP fusion proteins in N. *benthamiana* leaf epidermal cells. All the members of *Zm*CPKs were localized to the plasma membrane. eGFP was used as a positive control. Bars = 20 µm.

The expression site of the protein is closely related to its physiological function. Therefore, nine maize CPKs were fused with green fluorescent proteins regarding their subcellular localization in tobacco leaves. Among the tested *Zm*CPKs, *ZmCPK17*, 22, 28, and 38 were predicted to have a myristoylation motif, while ZmCPK14, 15, 26, 36, and 37 do not have myristoylation motif. In addition to the N-myristoylation motif, the palmitoylation site is also important for the membrane localization of CPK proteins. In this experiment, the non-myristoylated *Zm*CPKs were predicted to have palmitoylation sites. To verify the subcellular localization of these CPKs, eGFP fusion of these proteins was transiently expressed in tobacco leaves. The subcellular localization was analyzed by laser confocal microscopy. As shown in Figure 3, all nine *Zm*CPKs were localized to the cell membrane.

### 2.3. Interaction Between ZmCPK Family and A Subclass ZmPP2C Family

**Figure 4:**
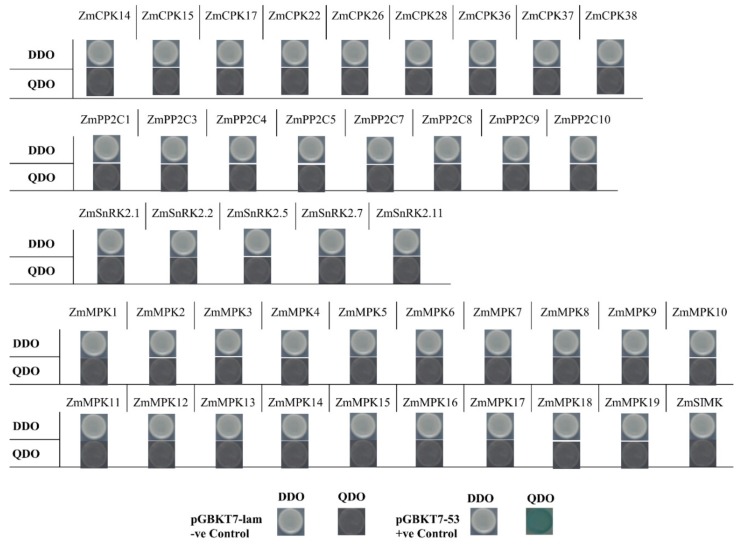
Self-activation detection of *Zm*CPK, *Zm*PP2C, *Zm*SnRK2, and *Zm*MPK family members for yeast two-hybrid assay. pGBKT7-lam and pGBKT7-53 were used as a negative and positive control, respectively. DDO medium = −Leu/−Trp, QDO = −Leu/−Trp/−His/−Ade/ + X-α-Gal.

Prior to the yeast two-hybrid assay, all recombinant vectors involved in this experiment were first tested by a self-activation assay, in which the gene fragment was inserted into the vector pGADT7, and the pGBDT7 empty vector was co-transformed into yeast strain Y2HGold, and the gene fragment was inserted. The recombinant vector pGBDT7 was co-transformed with the pGADT7 empty vector into the yeast strain Y2HGold, and the growth target was used to observe whether the inserted target fragment has the function of activating the yeast two-hybrid system alone (Figure 4). All the vectors of the experiment had no self-activation effect. False positive results caused by self-activation are excluded.

**Figure 5:**
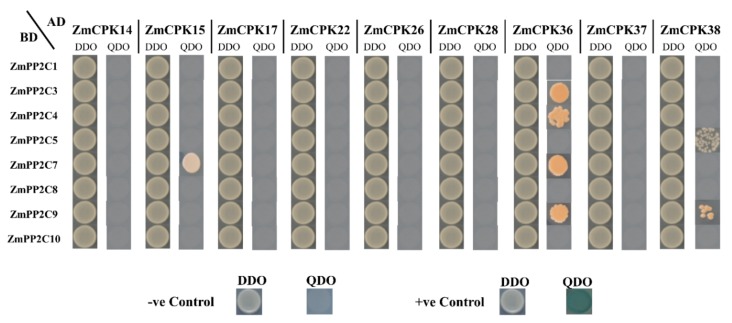
Y2H interaction assay between CPK and clade A *Zm*PP2C members. DDO medium lacking Leu/Trp, QDO medium lacking Leu/Trp/His/Ade with X-α-Gal. pGBKT7-Lam and pGBKT7-53 were used as −ve and +ve control, respectively. Pseudo interacting pairs grown on QDO are in brown color due to adenine deficiency and have no effect of X-α-Gal on them, indicating that these could be false positives.

There were 72 yeast double-hybrid interactions between nine *Zm*CPK genes and eight members of class A of *Zm*PP2C. *ZmPP2C1*/3/4/5/7/8/9 and 10 were tested in these interactions in different ways and combinations (Figure 5). Seven combinations (*Zm*CPK15×*Zm*PP2C7, *Zm*CPK36 × *ZmPP2C3*/4/7and 9 and *ZmCPK38* × *ZmPP2C5* and 9) showed possible interaction by growing on a selective QDO (−Leu/−Trp/−His/−Ade, X-α-Gal) medium, but had no effect of X-α-Gal application, which raised a question on the authenticity and strength of these interactions, which could be due to inaccessibility of prey protein to the UAS region that is related to Gal4 binding site in rare cases. In this case, we needed to confirm the interaction using additional techniques, e.g., coimmunoprecipitation (coIP) or Bimolecular fluorescence complementation (BiFC). To confirm the interaction between the pairs of possibly interacted proteins in yeast, we performed BiFC in tobacco leaves. The results of BiFC confirmed that the interaction showed by pairs in Y2H were false positives. We replicated the BiFC experiment three times to exclude any mistake and found negative results, indicating that there is no interaction between *Zm*CPKs and *Zm*PP2Cs in tobacco leaves.

### 2.4. Interaction of the ZmCPK Family and the ZmSnRK2 Family

There are 11 members in the *Zm*SnRK2 subclass. In this study, yeast two-hybrid assay was carried out with five *Zm*SnRK2s and nine *Zm*CPKs of maize. Based on the predicted interactions of the BrCPK-BrSnRK superfamily in Chinese cabbage [43], we used different combinations to verify the CPK-SnRK interaction in maize.

**Figure 6:**
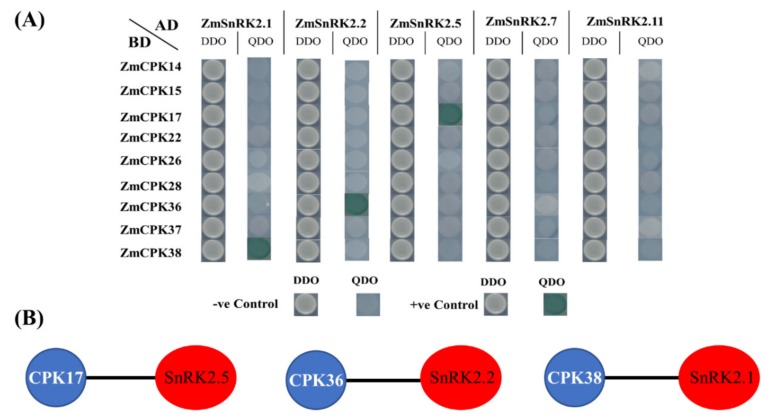
Y2H assay between *Zm*CPK and *Zm*SnRK2s. (**A**) Interaction was determined by the colony growth of the yeast strain Y2HGold co-transformed with these indicated plasmid combinations on DDO medium lacking Leu/Trp, QDO medium lacking−Leu/−Trp/−His/−Ade with X-α-Gal, pGBKT7-Lam and pGBKT7-53 were used as −ve and +ve controls, respectively. (**B**) Interactome of *Zm*CPK and *Zm*SnRK2 family members. The data are compiled from results presented in **A**.

Yeast two-hybrid experiments were performed between *Zm*SnRK2 and nine *Zm*CPKs in 42 combinations. Out of all these combinations, only three combinations showed interactions. Those combinations *wereZmCPK38* × *ZmSnRK2*.1, *ZmCPK36* × *ZmSnRK2*.2, and *ZmCPK17* × *ZmSnRK2*.5 (Figure 6). All these combinations showed interaction with each other as they grew in the form of blue dots on QDO medium.

### 2.5. Physical Interaction Between ZmCPK and ZmMPK Gene Families

**Figure 7:**
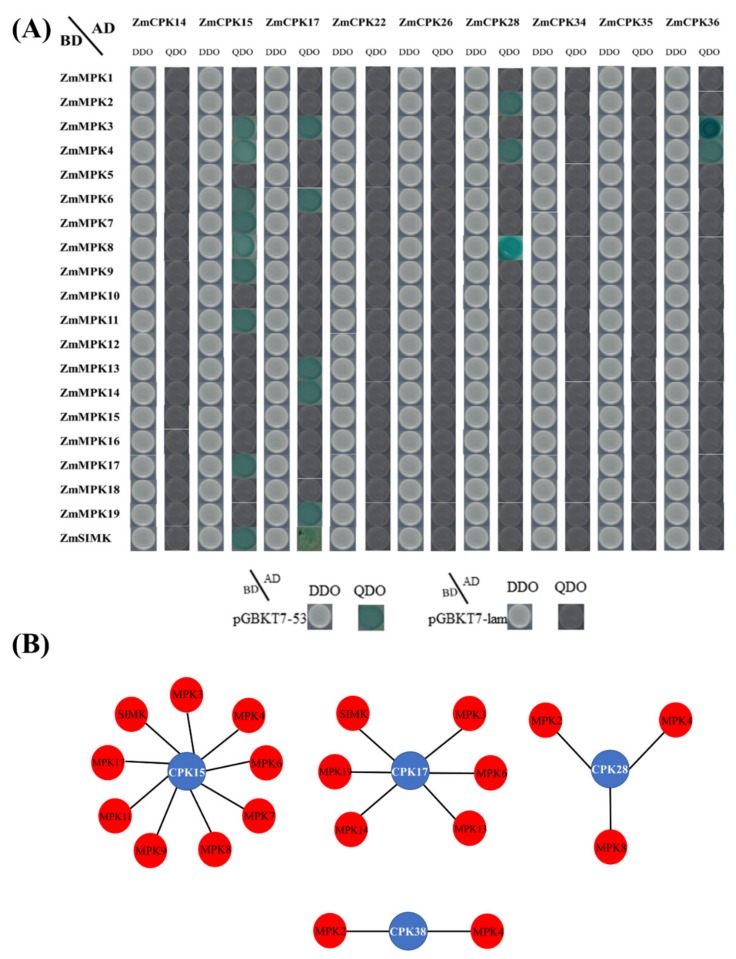
Y2H interaction assay between *Zm*CPK and *Zm*MPK members. (**A**) Interaction was determined by the colony growth of the yeast strain Y2HGold co-transformed with these indicated plasmid combinations on DDO medium lacking Leu/Trp and QDO medium lacking Leu/Trp/His/Ade with X-α-Gal. pGBKT7-Lam and pGBKT7-53 were used as −ve and +ve controls, respectively. (**B**) Interactome of *Zm*CPK and *Zm*MPK family members. The data were compiled from results presented in A.

In order to test the intersection of the calcium signaling pathway and MAPK signaling pathway, we tested the interaction between family members of *Zm*CPK and *Zm*MPK. Our results showed that there is a strong interaction between these two family members, as they activated in response to the same stimuli. In this study, nine members were cloned and constructed into a pGADT7 vector, and yeast two-hybrid assay was carried out with 20 members of *Zm*MPK of maize. *ZmCPK15*,17,28 and 38 interacted with *Zm*MPK family members in different combinations. Interactional pairs were *ZmCPK15* × *ZmMPK3*/4/6/7/8/9/11/17/*SIMK*, *ZmCPK17* × *ZmMPK3*/6/13/14/19/*SIMK*, *ZmCPK28* × *ZmMPK2*/4/8, and *ZmCPK38* × *ZmMPK3*/4 (Figure 7).

## 3. Discussion

### 3.1. Expression Profiles of ZmCPKs

An increasing body of evidence has shown that CPKs regulate ABA signal transduction in plants [11,26,28]. *ZmCPK17* was expressed at high levels in ABA treatment, but downregulated in calcium treatment, which indicated that these two treatments have antagonistic effects on the expression of *ZmCPK17* in leaves, while in roots, they showed the same trend but were lower in transcription levels, as described in an earlier study [44]. *ZmCPK14*, 22, and 37 showed downregulation in leaves under ABA treatment and upregulation in calcium treatment, depicting the sensitivity of these genes towards calcium and ABA treatments. *ZmCPK15* belongs to group A of the *Zm*CPK family and is closely related to *AtCPK4* and *AtCPK11* [45]. Biphasic fluctuation in the expression of *ZmCPK15* under calcium and ABA treatments, indicating its role in defense mechanism through increasing the production of superoxide dismutase (SOD) and ascorbate peroxidase (APX) in maize protoplasts [41]. *ZmCPK26* and *ZmCPK28* showed slight fluctuation under these treatments, indicating a possible role similar to their ortholog in rice, and suggesting that these two genes are not sensitive to calcium treatment or their affinity towards calcium is not enough for expression alterations as reported in *Arabidopsis* [46,47]. This minute fluctuation in expression time-course analysis showed that calcium treatment and ABA treatment led to a significant increase and decrease of the expressions of these members, indicating their positive and negative roles in the downstream signaling process.

Given the expression pattern of the genes, all the genes have a heartbeat-like expression pattern at different time points. We can predict per expression levels of these genes that the first hour is the most important after abiotic stress treatments, i.e., ABA and CaCl_2_, and these findings could be useful for researchers for detailed functional analysis.

### 3.2. Sub-Cellular Localization of CPK Genes in Maize

In plants, CPKs are widely distributed, having ubiquitous expression in roots, flowers, leaves, and siliques, etc. Some CPKs are expressed in multiple places, while others are specific with respect to their expression. [48,49,50] The widespread distribution of calcium-dependent protein kinases (CPKs) in plants indicates that they play significant roles in signaling transduction pathways by activating numerous substrates [51]. To examine the subcellular localizations of *Zm*CPKs, we used translational fusions with (GFP) green fluorescence protein. All the selected *Zm*CPKs were located in a plasma membrane (Figure 3). Most of the members of CPKs have predicted a N-myristoylation motif (asite with a gly residue at position 2), which is involved in membrane targeting. This co-translational acylation is irreversible and involves a second post-translational signal, such as reversible palmitoylation sites (a motif with cys residue at 4/5 position) to retain the membrane association [30,52]. *OsCPK18* showed that the N-terminus myristoylation site is vital for plasma membrane targeting [53]. *NtCPK5* lost its plasma membrane localization after three mutations (Gly2Ala or Cys4Ala or a double mutant of Gly2Ala/Cys4Ala) and showed distribution throughout the cell, suggesting the importance of N-myristoylation and palmitoylation sites for CPKs’ plasma membrane localization. [8,54] Supportively, *AtCPK34* and *AtCPK2* were localized on the plasma membrane, but after point mutation at myristoylation/palmitoylation sites, abolished the plasma membrane localization and caused cytoplasmic distribution [55]. In soybeans, *GmCPK3* and GmCPK31 had N-Myristoylation sites and were localized in the plasma membrane of *Arabidopsis* mesophyll protoplasts [56]. Another biochemical study, which was in line with our study, revealed that CPK is highly Ca2+ sensitive and associated with plasma membranes [47]. As calcium-dependent protein kinases take part in various aspects of plant biology, from transcription to ion transport [57], the localization of these *Zm*CPKs indicated their possible role in signal transduction or ion transportation in or out of the cell.

### 3.3. Interaction Analysis of ZmCPKs, ZmPP2Cs, and ZmSnRKs

In plants, protein-protein interactions are influenced by many factors, such as tissue and organ specificity, stress response expression patterns, subcellular localization, protein-to-protein preference, and phosphorylation levels, etc. [58].

*Zm*CPK, *Zm*PP2C, and *Zm*SnRK family members are numerous, their expression patterns and subcellular localizations are different, and interactions show variability in different crops, resulting in the complexity of the upstream calcium and ABA signal network of maize [59]. This diversity and complexity are also a favorable regulation mechanism for maize to cope with the external adverse environment. In ABA signaling, members of the PP2C and SnRK family were found to have interactions in maize [60], and in contrast to our study, members of PP2C and CPK were found to interact in canola [61]. In this study, possible points of interaction between Calcium and ABA signaling were studied in maize. In contrast to the results in *Brassica napus* and *Arabidopsis*, selected candidates of *Zm*CPK and *Zm*PP2C had no interaction in maize, suggesting that they may or may not be involved in other cellular functions [61,62]. The SnRK2 family is the major kinase involved in ABA signaling, and is classified into two subclasses of SnRK2a (*ZmSnRK2*.1/2.2/2.3/2.8/2.10) and SnRK2b (*ZmSnRK2*.4/2.5/2.6/2.7/2.11) according to the degree of enrichment of its C-terminal amino acids [63,64,65]. Among them, the SnRK2a subclass was confirmed to be mainly involved in the binding to PP2C and the transmission of ABA signals [66,67,68]. Along with the interaction and phosphorylation of other substrates, two kinases can phosphorylate each other, or they could have a common substrate [67]. Our results suggested that two kinases (CPKs and SnRKs) can interact with each other in a way that may activate downstream signaling. In the present study, both the *Zm*SnRK2a and *Zm*SnRK2b subclasses were found to interact with *Zm*CPK. Subcellular localization of SnRK2s [60] and our results of subcellular localization support these results of Y2H interactions. It implies that ZmSnRK2 members might be the intersectional point of calcium and ABA signal transmission. Further functional dissection of these individual interactions between *Zm*CPKs and *Zm*SnRKs will be needed to completely understand their specific role in the growth and development of maize.

### 3.4. Interaction of Calcium and MAPK Signaling Pathways

Plant protein interactions are controlled by many factors, including stress and tissue or organ specificity. Traditional MAPK cascade recognizes MAPK kinase as the only kinase for signal transmission in yeast, animal, and plant systems. Efforts have been made to study calcium/CPK and MAPK signaling in plants, but the crosstalk between these important kinase signaling pathways have not been studied. Previous studies suggest that these two pathways work parallel to each other without having direct crosstalk in *Arabidopsis* and tobacco [39]. In this study, interactional analysis of CPK and MAPK members in maize indicated that the calcium signaling pathway can directly influence the MAPK signaling mechanism through CPKs. Our results suggested that *Zm*CPK 15, 17, 28 and 38 make direct contributions to the crosstalk between calcium and MAPK signaling mechanisms in rice as reported by [42]. Generally, MAPKs are characterized by the conserved TXY motifs in their T-loop and activated by dual phosphorylation. *OsCPK18* (an ortholog of *ZmCPK38*) interacted with *OsMPK5* (an ortholog of *ZmMPK4*) and was identified as activating OsMPK5 by phosphorylating two threonine residues without affecting the phosphorylation of conserved TXY (TEY & TDY) motifs [42]. This scheme may be followed in maize, but it is difficult to determine the interactional activity from the primary amino acid sequence, because it largely depends upon conformational diversity. Our results implied that the crosstalk between calcium and MAPK signaling is not only carried out indirectly, but also directly, through the physical interaction of CPK and MAPK in maize. Further intensive research on the function of the CPK-MAPK complex will help us to explore the roles of these individual complexes and improve our understanding of crosstalk between these pathways in plant growth and development, as well as responses in biotic and abiotic stresses. These findings suggest that MAPKs are not only phosphorylated by MAP kinase kinase (MKK) but that plants might evolve another pathway to phosphorylate MAPKs via CPKs.

## 4. Materials and Methods

### 4.1. Plant Materials and Growth Conditions

The seeds of the model maize inbred line B73 were sterilized, germinated in petri dishes, and transplanted into 1/4 concentration Hoagland medium for hydroponic culture, and the nutrient solution was replaced once every 3–4 days. The culture temperature was 18–25 °C, the photoperiod was 12 h (light)/12 h (dark), and the light intensity was 400 μmol/s·m^2^.

### 4.2. ABA/CaCl_2_ Treatment and RNA Extraction

As described in the literature, 100uM ABA and 10 mM CaCl_2_ can significantly stimulate and mimic stress responses [41,60,69,70,71]. At the three-leaf stage, plants were treated with ABA and CaCl_2_ by soaking roots in nutrient solution with 100 μM (±) ABA ((Sigma-Aldrich, Foster City, CA, USA)) and 10 mM CaCl_2_. After 0 (Control), 6, 12, 24 and 48 h of ABA treatment and 0 (Control), 10, 20, 30, 60, 90, 120 and 240 min of calcium chloride treatment, roots and leaves were sampled and three biological replicates were set for each set of samples. Roots and leaves were sampled separately and frozen at −80 °C immediately after collection. RNAiso Plus (TaKaRa, Kusatsu, Japan) and the PrimeScript™II1st strand cDNA synthesis kit (Takara, Kusatsu, Japan) were used for total RNA extraction and reverse transcription for cDNA synthesis, respectively.

### 4.3. Quantitative Real-Time PCR

The Primer-BLAST tool (http://www.ncbi.nlm.nih.gov/tools/primer-blast) was used to design gene-specific primers to amplify a 100–300 bp fragment of each selected member of the *Zm*CPK gene family (Supplementary Table S1). The cDNA samples prepared above were used as templates for SYBR green-based qRT-PCR analysis. Each reaction mixture (10 μL) contained SYBR Premix Ex Taq II (Tli RNaseH Plus) 5 μL, F/R primer 0.5 μL, diluted 1 μL of cDNA, and ddH2O supplemented to 10 μL. The thermal cycling conditions were 95 °C for 30 sec followed by 39 cycles of 95 °C for 5 s, 55–60 °C for 30 s and 72 °C for 15 s. Referring to Lin et al. (2014) [72], the ZmGAPDH gene was used as an internal control. The 2^−ΔΔ*C*T^ method of the CFX Manger™ software version 2.0 (Bio-Rad, Philadelphia, PA, USA) was used to normalize the differential gene expression among the multiple internal controls and the target genes [72].

### 4.4. Subcellular Localization

Gene-specific primers were used to amplify the targeted coding sequences of nine *Zm*CPKs with appropriate restriction sites and without terminal codons from cDNA samples (Supplementary Table S2). Amplified fragments were ligated in-frame to the 5’-terminus with the expression vector pCambia2300-eGFP. Constructed plasmids were infiltrated into abaxial air space of three-week-old *N. benthamiana* leaves with the help of transformed *Agrobacterium* strain GV3101. Infiltrated parts of the leaves were marked and fluorescence was observed under the confocal laser scanning microscope model A1 (Nikon, Tokyo, Japan) after 48 h of infiltration.

### 4.5. Yeast Two‑Hybrid Assay

The complete coding sequences of 9 *Zm*CPK family members from different phylogenetic groups, 8 *Zm*PP2C family, 5 genes of *Zm*SnRK family and 20 genes from the *Zm*MPK family (Figure 1) were amplified by using gene specific primers from the cDNA samples (primer information in Supplementary Table S3). These amplified fragments were inserted into bait (pGBKT7) and pray (pGADT7) vectors of the yeast two-hybrid assay after sequence confirmation by sequencing. Yeast strain Y2HGold was co-transferred in all possible combinations of pGBKT7-*Zm*CPK14, 15, 17, 22, 26, 28, 36, 37, 38 with pGADT7-*ZmSnRK2*.1, 2.2, 2.5, 2.7, 2.11, and pGADT7-*ZmCPK14*, 15, 17, 22, 26, 28, 36, 37, and 38 with pGBKT7-*ZmPP2C1*, 3–5, and 7–10, pGBKT7-*ZmMPK1*-19, and ZmSIMK (primer information in Supplementary Tables S4 and S5, MAPK identifiers in Supplementary Table S8), according to the protocol provided by Yeastmaker^TM^ Yeast Transformation System 2 (Clontech, Japan). pGBKT7-53 and pGBKT7-lam were used as positive and negative controls, respectively. To exclude the possible autoactivation of *Zm*CPK members, a control experiment was carried out by co-transformation of loaded bait and prey plasmids with empty prey and bait plasmids, respectively. After screening on solid DDO (−Leu/−Trp) medium for 3 days at 30 °C, selected monoclones were inoculated in liquid DDO (−Leu/−Trp) medium until OD600 = 0.7−1.0. These cultures were inoculated on QDO (−Leu/−h−His/-Ade/ + X-α-Gal) medium after ten-fold dilution. Results were observed after 4–5 days of incubation at 30 °C.

### 4.6. Bimolecular Fluorescence Complementation

Coding sequences of *ZmCPK15*/36/38 and *ZmPP2C3*/4/5/7/9 were amplified by using two sets of primers (Supplementary Tables S6 and S7) and inserted into pSAT6-nEYFP-N1_(E2913) and pSAT6A-cEYPF-N1_(pE3086) yellow fluorescent protein plasmids without stop codon, respectively. These plasmids were transformed into Agrobacterium tumefaciens strain GV3101. As described by Wang et al. 2018 [60], transformed A. tumefaciens strains loaded with expression plasmids pSAT6-CPKs- nEYFP and pSAT6A-*Zm*PP2Cs-cEYPF were co-infiltrated into the abaxial air space of 22 days old *Nicotiana benthamiana* leaves at OD600 = 0.7:0.7. The epidermal cells of the tobacco leaves near the infiltrated sites were observed for fluorescence after 36 h of the co-infiltration under confocal laser scanning microscope model A1 (Nikon, Tokyo, Japan).

## 5. Conclusions

In this study, 9 members of the *Zm*CPK family were amplified from different groups on the basis of phylogenetic analysis. qRT-PCR analysis showed that most of the *Zm*CPK genes are responsive to both ABA and CaCl_2_ treatments inferred by heat maps. Furthermore, subcellular localization of the candidate genes provided preliminary information for future investigation of the CPK family in maize. Moreover, three members of the *Zm*CPK family interacted with the members of *Zm*SnRK2 family that are active members in the core ABA signaling cascade. Four *Zm*CPK members were found to interact with thirteen *Zm*MPK members, depicting the complexity of signaling networks. The current investigation lays the groundwork for a functional characterization of the *Zm*CPK gene family to improve our understanding of complex signaling pathways.

## Supplementary Materials

Supplementary materials can be found at https://www.mdpi.com/1422-0067/20/24/6173/s1.
