# Supplementary material for: Expression, Subcellular Localization, and Interactions of CPK Family Genes in Maize
Source: Int J Mol Sci. 2019 Dec 6;20(24):6173. doi: 10.3390/ijms20246173 (PMC6940914; doi:10.3390/ijms20246173)
Supplement: Supplementary file 1 [file ijms-20-06173-s001.pdf]

Supplementary Table S1. Primers used for the Real Time-PCR of *ZmCPK* gene family.

Name	Identifier	qPCR Primers (5'-3')
ZmCDPK14	GRMZM2G035843	**F:** ACTTGGTTTCGGCATTCTCATT<br>**R:** CCTCATCATCGCTGTGAACTCG
ZmCDPK15	GRMZM2G047486	**F:** TACCGCATCGGCAAGAAACT<br>**R:** CTCACTGTAGTGGCCCTTGG
ZmCDPK17	GRMZM2G463464	**F:** TTTAGTCCGTAAGATGCTTATCCG<br>**R:** AATAGGCTTATCAGGTGCGACA
ZmCDPK22	GRMZM2G058305	**F:** ATGAGGTGTGTCGTGTGGTCTA<br>**R:** GCATTACTTTTCGCCTGGTAT
ZmCDPK26	GRMZM2G154489	**F:** TTGCGACCAAGGAGGAGAAT<br>**R:** TTGCGACCAAGGAGGAGAAT
ZmCDPK28	GRMZM2G168706	**F:** CGTCCTCCCTCCAACTCCAAGA<br>**R:** TGCGTCGATGAGACAAATCAAGAG
ZmCDPK36	GRMZM2G028086	**F:** TACACGGTAGCCCGATAACA<br>**R:** CAAGCGAAGAATACAAAGCAA
ZmCDPK37	GRMZM2G099425	**F:** TGTTGATGCCGTGATTGTATTT<br>**R:** ATGGGTCCACAGAAGTTAAGAA
ZmCDPK38	GRMZM2G365035	**F:** CAGGGCCAGAATCCGATGTT<br>**R:** ATGCCTGTGCAGCAGTTAGT

Supplementary Table S2. Primers used for subcellular localization of *ZmCPK* gene family

Accession Number	Gene	Sequence
GRMZM2G035843	*ZmCPK14*	F: 5'-gagaggacagggtaccATGCAGCCGGACCCGAGCGGGA-3'<br>R: 5'-ttgctcaccatggtactagtGGTTTTGCTGGGATTCAAGAGT-3'
GRMZM2G047486	*ZmCPK15*	F: 5'-gagaggacagggtaccATGCAGCCGGACCCGAGCGGGA-3'<br>R: 5'-ttgctcaccatggtactagtGGTTTTGCTGGGATTCAAGAGT-3'
GRMZM2G463464	*ZmCPK17*	F: 5'-gagaggacagggtaccATGCAGCCGGACCCGCAAGGCC-3'<br>R: 5'-ttgctcaccatggtactagtCTAGGTCTCGGTGGGCTTCAGG-3'
GRMZM2G058305	*ZmCPK22*	F: 5'-gagaggacagggtaccATGGGCGGCCGCGCCTCCCGCC-3'<br>R: 5'-ttgctcaccatggtactagtTTAAAACATGCGCCTCCGATTT-3'
GRMZM2G154489	*ZmCPK26*	F: 5'-gagaggacagggtaccATGGGGCAGTGTTGCAGCAGAG-3'<br>R: 5'-ttgctcaccatggtactagtCTTGAGCCTCATTGGCTGCTGC-3'
GRMZM2G168706	*ZmCPK28*	F: 5'-gagaggacagggtaccATGGGGCAGTGTTGTAGCAGAG-3'<br>R: 5'-ttgctcaccatggtactagtCTTGAGCCTCATTGGCTGCTGC-3'
GRMZM2G028086	*ZmCPK36*	F: 5'-gagaggacagggtaccATGGGCAACTGCTGCGTGACGC-3'<br>R: 5'-ttgctcaccatggtactagtCTACCGTGTACTCGTCATCTGC-3'
GRMZM2G099425	*ZmCPK37*	F: 5'-gagaggacagggtaccATGGGCAACTGCTGCGTAACAC-3'<br>R: 5'-ttgctcaccatggtactagtCTGCGTACTCTCCATCTGCAAC-3'
GRMZM2G365035	*ZmCPK38*	F: 5'-gagaggacagggtaccATGGGCCTCTGCTCCTCCTCCA-3'<br>R: 5'-accatggtactagtgtcgacCTATGACTTGCGAACACCCCTA-3'

Supplementary Table S3. Primers for cloning *ZmCPK, ZmPP2C and ZmSnRK* family genes

Gene ID	Gene	Sequence
GRMZM2G035843	*ZmCPK14*	F: 5'-CTAATTCCATATGATGCAGCCGGACC-3'<br>R: 5'-GCGAGTCGACCTAGGTTTTGCTG-3'
GRMZM2G047486	*ZmCPK15*	F: 5'-GGAATTCCATATGATGCAGCCGGAC-3'<br>R: 5'-AGACGTCGACCTAGGTTTTGCTGG-3'
GRMZM2G463464	*ZmCPK17*	F: 5'-CGGGATCCATGCAGCCGGACC-3'<br>R: 5'-AACTGCAGCTAGGTCTCGGTGGGC-3'
GRMZM2G058305	*ZmCPK22*	F: 5'-CGAATTCATGGGCGGCCGCG-3'<br>R: 5'-CGGGATCCTTAAAACATGCGCCTCCGATTTG-3'
GRMZM2G154489	*ZmCPK26*	F: 5'-GGAATTCATGGGGCAGTGTTGCAGC-3'<br>R: 5'-ACGCGTCGACCTACTTGAGCCTCATT-3'

GRMZM2G168706	*ZmCPK28*	F: 5'-CGGAATTCATGGGGCAGTGTTGTAG-3'<br>R: 5'-GCGAGTCGACCTACTTGAGCCTCA-3'
GRMZM2G028086	*ZmCPK36*	F: 5'-CTAATTCCATATGATGGGCAACTGCT-3'<br>R: 5'-CGGAATTCCTACCGTGTACTCGTC-3'
GRMZM2G099425	*ZmCPK37*	F: 5'-CCGGATCCATGGGCAACTGCT-3'<br>R: 5'-GCCTGCAGCTACTGCGTACTCT-3'
GRMZM2G365035	*ZmCPK38*	F: 5'-CGAATTCATGGGCCTCTGCTCCTCC-3'<br>R: 5'-ACGCGTCGACCTATGACTTGCGAACA-3'
GRMZM2G102255	*ZmPP2C1*	F: CCCATATGATGACCCACCACTACCCCCCGA<br>R:CGGGATCCTTATGCTCTGCTCTTGATCTTCCTA
GRMZM2G059453	*ZmPP2C3*	F: TGAATTCATGGCCGAGATCTGCTGCGAG<br>R: GGGATCCTTCGCTCCGAGAATCATATGC
GRMZM2G177386	*ZmPP2C4*	F: CCCATATGATGGCGGCGGCGATGTGCGTGG<br>R: CGGAATTCTCAAATATCGGTCCCCAATCGATCT
GRMZM2G437575	*ZmPP2C5*	F: CCCATATGATGGCATGGTTCCTGCAAGTGG<br>R: CGGAATTCTCAGCTCAGCTCTACTGTGATGGAC
GRMZM2G383807	*ZmPP2C7*	F: CGGAATTCATGGAGGACCTCGCCCC<br>R: GCCCTAGGTTATGTTCTGCTCTTGAACTTTCTA
GRMZM2G019819	*ZmPP2C8*	F: CCCATATGATGACCAGCGCCGGCGTCAAC<br>R: CGGAATTCTCACTCCCTGTGCTTGAGGTCAACG
GRMZM2G122228	*ZmPP2C9*	F: CCCATATGGAAGAGATGAGCAGCAGTGA<br>R: CGGAATTCCTACAGACTACGTCCTACCATTCTG
GRMZM2G001243	*ZmPP2C10*	F: CCCATATGATGGCGGCGGCGGCGATCTGC<br>R: CGGAATTCCTATAATCTACATCCCACTTAAAAA
GRMZM2G035809	*ZmSnRK2.1*	F: CCCATATGGGATGGAGCGGTACGAGGTGAT<br>R: CGGGATCCCGCCTCACACAGTCACACTGCACA
GRMZM2G056732	*ZmSnRK2.2*	F: CCCATATGGGATGGAGAGGTACGAGGTGAT<br>R: CGGGATCCCGTCACAAGGCACATACAAAGTC
GRMZM2G110908	*ZmSnRK2.5*	F: CCCATATGGGATGGACAAGTACGAGCCC

		R: CCCTCGAGGGATACTAAGAGTCAGATTTGG
GRMZM2G155593	*ZmSnRK2.7*	F: CGCATATGGAGAAACACGAGCTATTGA<br>R: ATGAATTCTTAACAAGAAAGATGAAAC
GRMZM2G063961	*ZmSnRK2.11*	F: CCCATATGGGATGGACAAGTACGAGGCT<br>R: CGGGATCCCGAGGCAGCATTTAGATGTG

Supplementary Table S4 Primers for pGADT7 vectors construction of *ZmCDPK* genes

Gene	Sequence	Restriction Site	Buffer	Restriction Temperature	Annealing Temperature
ZmCPK14	F: 5'-GGAATTCCATATGATGCAGCCGGAC-3'<br>R: 5'-TCCCCCGGGCTAGGTTTTGCT-3'	*Nde* I<br>*Sma* I	1×T+BSA	30 ℃	61.2 ℃<br>61.5 ℃
ZmCPK15	F: 5'-AGAATTCCATATGATGCAGCCGGAC-3'<br>R: 5'-TAACCCGGGCTAGGTTTTGCT-3'	*Nde* I<br>*Sma* I	1×T+BSA	30 ℃	59.6 ℃<br>57.6 ℃
ZmCPK17	F: 5'-CGAATTCATGCAGCCGGACC-3'<br>R: 5'-CGGGATCCCTAGGTCTCGGTG-3'	*Eco* RI<br>*Bam* HI	1×K	30 ℃	59.5 ℃<br>63.4 ℃
ZmCPK22	F: 5'-CGAATTCATGGGCGGCCGCG-3'<br>R: 5'-CGGGATCCTTAAAACATGCGCCTCCGATTTG-3'	*Eco* RI<br>*Bam* HI	1×K	30 ℃	63.6 ℃<br>64.9 ℃
ZmCPK26	F: 5'-CGAATTCATGGGGCAGTGTTG-3'<br>R: 5'-CGGGATCCCTACTTGAGCCTCAT-3'	*Eco* RI<br>*Bam* HI	1×K	30 ℃	57.6 ℃<br>61.3 ℃
ZmCPK28	F: 5'-GGAATTCCATATGATGGGGCAGTG-3'<br>R: 5'-CGAATTCCTACTTGAGCCTCATTGGC-3'	*Nde* I<br>*Eco* RI	1×H	37 ℃	59.3 ℃<br>61.2 ℃
ZmCPK34	F: 5'-GGAATTCCATATGATGGGCAACTG-3'<br>R: 5'-CGAATTCCTACCGTGTACTCGTCAT-3'	*Nde* I<br>*Eco* RI	1×H	37 ℃	57.9 ℃<br>59.6 ℃
ZmCPK35	F: 5'-CGAATTCATGGGCAACTGCTGC-3'<br>R: 5'-AGGGATCCCTACTGCGTACTCTCC-3'	*Eco* RI<br>*Bam* HI	1×K	30 ℃	59.5 ℃<br>63.0 ℃
ZmCPK36	F: 5'-GTAATTCCATATGATGGGCCTCTGCTCC-3'<br>R: 5'-CGAATTCCTATGACTTGCGAACACCCC-3'	*Nde* I<br>*Eco* RI	1×H	37℃	62.5 ℃<br>62.6 ℃
ZmSnRK2.1	F: 5'-CCCATATGGGATGGAGCGGTACGAGGTGAT-3'<br>R:5'-CGGGATCCCGCCTCACACAGTCACACTGCACA-3'	*Nde* I<br>*Bam* HI	1×K	30 ℃	58.5 ℃<br>62.0 ℃
ZmSnRK2.2	F:5'-CCCATATGGGATGGAGAGGTACGAGGTGAT-3'<br>R:5'-CGGGATCCCGTCACAAGGCACATACAAAGTC-3'	*Nde* I<br>*Bam* HI	1×K	30 ℃	59.5 ℃<br>61.0 ℃
ZmSnRK2.5	F:5'-CCCATATGGGATGGACAAGTACGAGCCC-3'<br>R:5'-CCCTCGAGGGATACTAAGAGTCAGATTTGG-3'	*Nde* I<br>*SmlI*	1×K	30 ℃	57.5 ℃<br>63.0 ℃
ZmSnRK2.7	F: 5'-GCATATGGAGAAACACGAGCTATTGA-3'<br>R:5'-TGAATTCTTAACAAGAAAGATGAAAC-3'	*Nde* I<br>*Eco* RI	1×H	37 ℃	58.9 ℃<br>59.6 ℃
ZmSnRK2.11	F: 5'-CCCATATGGGATGGACAAGTACGAGGCT-3'<br>R: 5'-CGGGATCCCGAGGCAGCATTTAGATGTG-3'	*Nde* I<br>*Bam* HI	1×K	30 ℃	59.5 ℃<br>63.0 ℃

Supplementary Table S5 Primers for pGBKT7 vector construction of *ZmCDPK* genes

Gene	Sequence	Restriction sites	Buffer	Restriction temperature	Annealing temperature
ZmCPK14	F: 5'-CTAATTCCATATGATGCAGCCGGACC-3'<br>R: 5'-GCGAGTCGACCTAGGTTTTGCTG-3'	*Nde* I<br>*Sal* I	1×H	37 °C	68.9 °C<br>67.9 °C
ZmCPK15	F: 5'-GGAATTCCATATGATGCAGCCGGAC-3'<br>R: 5'-AGACGTCGACCTAGGTTTTGCTGG-3'	*Nde* I<br>*Sal* I	1×H	37 °C	61.2 °C<br>61.3 °C
ZmCPK17	F: 5'-CGGGATCCATGCAGCCGGACC-3'<br>R: 5'-AACTGCAGCTAGGTCTCGGTGGGC-3'	*Bam* HI<br>Pst I	1×K	30 °C	73.1 °C<br>73.8 °C
ZmCPK22	F: 5'-CGAATTCATGGGCGGCCGCG-3'<br>R: 5'-CGGGATCCTTAAAACATGCGCCTCCGATTTG-3'	*Eco* RI<br>*Bam* HI	1×K	30 °C	63.6 °C<br>64.9 °C
ZmCPK26	F: 5'-GGAATTCATGGGGCAGTGTTGCAGC-3'<br>R: 5'-ACGCGTCGACCTACTTGAGCCTCATT-3'	*Eco* RI<br>*Sal* I	1×H	37 °C	72.4 °C<br>72.8 °C
ZmCPK28	F: 5'-CGGAATTCATGGGGCAGTGTTGTAG-3'<br>R: 5'-GCGAGTCGACCTACTTGAGCCTCA-3'	*Eco* RI<br>*Sal* I	1×H	37 °C	68.4 °C<br>68.8 °C
ZmCPK34	F: 5'-CTAATTCCATATGATGGGCAACTGCT-3'<br>R: 5'-CGGAATTCCTACCGTGTACTCGTC-3'	*Nde* I<br>*Eco* RI	1×H	37 °C	65.0 °C<br>67.0 °C
ZmCPK35	F: 5'-CCGGATCCATGGGCAACTGCT-3'<br>R: 5'-GCCTGCAGCTACTGCGTACTCT-3'	*Bam* HI<br>*Pst* I	1×K	30 °C	68.7 °C<br>68.6 °C
ZmCPK36	F: 5'-CGAATTCATGGGCCTCTGCTCCTCC-3'<br>R: 5'-ACGCGTCGACCTATGACTTGCGAACA-3'	*Eco* RI<br>*Sal* I	1×H	37 °C	64.5 °C<br>62.8 °C
ZmPP2C1	F: 5'-CCCATATGATGACCCACCACTACCCCCCGA<br>R: 5'-CGGGATCCTTATGCTCTGCTCTTGATCTTCCTA	*Nde* I<br>*Bam* HI	1×K	30 °C	59.5 °C<br>63.0 °C
ZmPP2C3	F: 5'-TGAATTCATGGCCGAGATCTGCTGCGAG-3'<br>R: 5'-GGGATCCTTCGCTCCGAGAATCATATGC-3'	*Eco* RI<br>*Bam* HI	1×K	30 °C	62.6 °C<br>63.9 °C
ZmPP2C5	F: 5'-CCCATATGATGGCATGGTTCCTGCAAGTGG-3'<br>R: 5'-<br>CGGAATTCTCAGCTCAGCTCTACTGTGATGGAC-3'	*Nde* I<br>*Eco* RI	1×H	37 °C	64.0 °C<br>62.0 °C
ZmPP2C7	F: 5'-CGGAATTCATGGAGGACCTCGCCCC-3'<br>R: 5'-GCCCTAGGTTATGTTCTGCTCTTGAACTTTCTA-<br>3'	*Eco* RI<br>*StyI*	1×H	37 °C	59.0 °C<br>61.0 °C
ZmPP2C8	F: 5'-CCCATATGATGACCAGCGCCGGCGTCAAC-3'<br>R: 5'-<br>CGGAATTCTCACTCCCTGTGCTTGAGGTCAACG-3'	*Nde* I<br>*Eco* RI	1×H	37 °C	64.5 °C<br>63.0 °C
ZmPP2C9	F: 5'-CCCATATGGAAGAGATGAGCAGCAGTGA-3'<br>R: 5'-<br>CGGAATTCCTACAGACTACGTCCTACCATTCTG-3'	*Nde* I<br>*Eco* RI	1×H	37 °C	62.0 °C<br>64.0 °C
ZmPP2C10	F: 5'-CCCATATGATGGCGGCGGCGGCGATCTGC-3'<br>R: 5'-<br>CGGAATTCCTATAATCTACATCCCACTTAAAAA-3'	*Nde* I<br>*Eco* RI	1×H	37 °C	60.0 °C<br>62.0 °C

Supplementary Table S6. Primers for pSAT6A-cEYPF-N1 vector construction of clade A *ZmPP2C* genes

Gene	Accession Number	Sequence
ZmPP2C1	GRMZM2G102255	F: 5'-tttacgaacgatagagatctATGGACGACCTCACCGTGGGGG-3'<br>R: 5'-gactgcagaattcgaagcttTGCTCTGCTCTTGATCTTCCTAR-3'
ZmPP2C3	GRMZM2G059453	F: 5'-cgagctcaagcttcgaattcATGGCCGAGATCTGCTGCGAGG-3'<br>R: 5'-ctgcacgctgcccaggatccTATGCCCCGGCGGAGATCCACG-3'
ZmPP2C4	GRMZM2G177386	F: 5'-tttacgaacgatagagatctATGGCGGCGGCGATGTGCGTGG-3'<br>R: 5'-taccgtcgactgcagaattcTGAGTTGCTCTTGGCCTTCTTT-3'
ZmPP2C5	GRMZM2G437575	F: 5'-tttacgaacgatagagatctATGGTCGGCCGGATGGAGCGGC-3'<br>R: 5'-taccgtcgactgcagaattcGCAGCGGAAACGGATGACAATG-3'
ZmPP2C7	GRMZM2G383807	F: 5'-tttacgaacgatagagatctATGGAGGACCTCGCCCCGGGGG-3'<br>R: 5'-taccgtcgactgcagaattcTTATGTTCTGCTCTTGAACTTT-3'
ZmPP2C8	GRMZM2G019819	F: 5'-tttacgaacgatagagatctATGTCGGCGTCGCGGAGCGGGA-3'<br>R: 5'-taccgtcgactgcagaattcTTCACTTGACCCGCTATTTCTT-3'
ZmPP2C9	GRMZM2G122228	F: 5'-tttacgaacgatagagatctATGAGCAGCAGTGAGGCTAGCA-3'<br>R: 5'-taccgtcgactgcagaattcCGTCCTACCATTCTGTCTACCA-3'
ZmPP2C10	GRMZM2G001243	F: 5'-tttacgaacgatagagatctATGGCGGCGGCGGCGATCTGCG-3'<br>R: 5'-taccgtcgactgcagaattcCATCCCACTTAAAAAGGGGAAA-3'

Note: The lowercase and uppercase letters stand for sequences homologous to pSAT6A-cEYPF-N1_vector and the clade A *ZmPP2C* genes, respectively.

Supplementary Table S7 Primers for pSAT6-nEYFP-N1 vector construction of *ZmCPK* genes

Gene	Accession Number	Sequence
ZmCPK15	GRMZM2G047486	F: 5'-gatagccatggtccggactcagatctATGCAGCCGGACC-3'<br>R: 5'-ccgggcccgcggtaccgGTCTCGGTGGGC-3'
ZmCPK36	GRMZM2G028086	F: 5'-gatagccatggtccggactcagatctATGGGCAACTGCT-3'<br>R: 5'-ccgggcccgcggtacccCGTGTACTCGTC-3'
ZmCPK38	GRMZM2G365035	F: 5'-gatagccatggtccggactcagatctATGGGCCTCTGCTCCTCC-3'<br>R: 5'-ccgggcccgcggtACCTGACTTGCGAACA-3'

Note: The lowercase and uppercase letters stand for sequences homologous to pSAT6-nEYFP-N1_vector and *ZmCPK* genes, respectively.

Supplementary Table S8: Members of ZmMAPK family.

No.	Gene	Accession Number	No.	Gene	Accession Number
1	*ZmMPK1*	GRMZM2G123886	12	*ZmMPK12*	GRMZM2G062761
2	*ZmMPK2*	GRMZM2G062914	13	*ZmMPK13*	GRMZM2G163861
3	*ZmMPK3*	GRMZM2G017792	14	*ZmMPK14*	GRMZM2G131334
4	*ZmMPK4*	GRMZM2G053987	15	*ZmMPK15*	GRMZM2G306028
5	*ZmMPK5*	GRMZM2G020216	16	*ZmMPK16*	GRMZM2G034052
6	*ZmMPK6*	GRMZM2G089484	17	*ZmMPK17*	GRMZM2G374088
7	*ZmMPK7*	GRMZM2G002100	18	*ZmMPK18*	GRMZM2G122335
8	*ZmMPK8*	GRMZM2G048455	19	*ZmMPK19*	GRMZM2G00784
9	*ZmMPK9*	GRMZM2G135904	20	*SIMK*	GRMZM2G127141
11	*ZmMPK11*	GRMZM2G375975			
